# Strategy Selection and Outcomes of Tip-Detection in CTO-PCI: Intraplaque Tracking vs Antegrade Dissection and Re-Entry

**DOI:** 10.1016/j.jacadv.2026.103274

**Published:** 2026-09-22

**Authors:** Kazunori Yamaji, Kota Tanaka, Tomohiro Yamasaki, Yoshitomo Tsutsui, Kiwamu Sudo, Satoshi Watanabe, Mutsumi Iwamoto, Nobuaki Tanaka, Yasushi Koyama, Yoshitaka Iwanaga, Heitaro Watanabe, Yoshihiro Fukumoto, Atsunori Okamura

**Affiliations:** a Division of Cardiovascular Medicine, Department of Internal Medicine, Kurume University School of Medicine, Kurume, Japan; b Cardiovascular Center, Sakurabashi Watanabe Advanced Healthcare Hospital, Osaka, Japan

**Keywords:** antegrade dissection and re-entry, chronic total occlusion, intraplaque tracking, percutaneous coronary intervention, tip detection method

## Abstract

**Background:**

The tip-detection (TD) method expands antegrade options for chronic total occlusion (CTO) percutaneous coronary intervention, but criteria for selecting TD intraplaque tracking (TD-IPT) vs TD antegrade dissection and re-entry (TD-ADR) remain unclear.

**Objectives:**

The authors evaluated factors associated with the initially selected TD strategy and procedural outcomes.

**Methods:**

We retrospectively analyzed 73 TD-attempted CTO-percutaneous coronary intervention cases from November 2020 to December 2023 and classified them as TD-primary IPT (n = 37) or TD-primary ADR (n = 36). Procedural outcomes were evaluated in all cases, and secondary metrics in 69 successful cases.

**Results:**

TD success was 97.3% (36/37; 95% CI: 85.8%-99.9%) and 97.2% (35/36; 95% CI: 85.5%-99.9%), and final procedural success was 94.6% (35/37; 95% CI: 81.8%-99.3%) and 97.2% (35/36; 95% CI: 85.5%-99.9%) in the TD-primary IPT and TD-primary ADR groups, respectively. TD-primary ADR was selected more often for J-CTO score ≥2 (83.3% [30/36] vs 62.2% [23/37]; *P* = 0.043) and severe calcification (47.2% [17/36] vs 2.7% [1/37]; *P* < 0.001). Among successful cases, lesion crossing time was shorter with TD-primary IPT (75 [51-108] vs 93 [63-137] minutes; *P* = 0.046), whereas stent length was shorter with TD-primary ADR (38 [28-54] vs 46 [38-64] mm; *P* = 0.041). In multivariable models, severe calcification was associated with longer lesion crossing time, and bending ≥45° was associated with longer stent length.

**Conclusions:**

TD-primary ADR was preferentially selected for complex, calcified CTOs. Procedural efficiency appeared driven mainly by lesion characteristics rather than the initial TD strategy, supporting individualized TD strategy selection based on lesion morphology and technical feasibility.

Over the years, various techniques have been developed for percutaneous coronary intervention (PCI) of chronic total occlusions (CTOs). However, once an antegrade guidewire enters the subintimal space, re-entry into the true lumen can be difficult. The retrograde approach has markedly improved PCI success rates for CTOs that were previously challenging to cross with an antegrade strategy. Nevertheless, procedural failure was not uncommon when suitable collaterals for retrograde intervention were absent or when guidewire crossing remained difficult even with a retrograde approach.

In 2020, Okamura et al proposed the tip-detection (TD) method, in which an intravascular ultrasound (IVUS) catheter is advanced over a guidewire that has entered the subintimal space, and a second guidewire is then guided through the plaque toward the CTO exit under IVUS guidance, thereby improving outcomes of antegrade wiring.^1^, ^2^, ^3^ This technique is currently referred to as tip-detection intraplaque tracking (TD-IPT). However, guidewire guidance can be challenging in lesions with poor target visualization, such as heavily calcified CTOs. Conventional antegrade dissection and re-entry (ADR) using the Stingray re-entry system (Boston Scientific) is an established option for true-lumen re-entry in antegrade CTO-PCI, including in calcified lesions.^4^ However, this technique requires angiography-guided puncture into the true lumen and may have limitations in reliability. In this context, Okamura and Suzuki et al reported in 2022 the usefulness of tip-detection ADR (TD-ADR), which performs ADR under IVUS guidance using the TD method, and further improved the success rate of antegrade CTO intervention.^5^^,^^6^ Even when TD-IPT fails to achieve lesion crossing, conversion to TD-ADR can often facilitate successful true-lumen crossing. Along with advances in devices such as AnteOwl IVUS (Terumo Corporation) and the Conquest Pro 12ST guidewire (Asahi Intecc), the TD strategy is becoming an increasingly established technique.^7^, ^8^, ^9^

However, when performing TD, the decision of whether to proceed with TD-IPT or TD-ADR still largely depends on operator judgment, and no clear criteria have been established. Therefore, we investigated factors associated with selection of the TD strategy and evaluated differences in procedural outcomes according to TD-IPT vs TD-ADR.

## Methods

### Study population

This was a single-center, retrospective observational study evaluating TD strategy selection and procedural outcomes according to the initially selected TD strategy. We included all CTO-PCI procedures performed at Sakurabashi Watanabe Advanced Healthcare Hospital between November 2020 and December 2023. Among these, we retrospectively identified cases in which the TD strategy was used for CTO crossing. Cases in which PCI was performed for multiple CTO lesions in a single session were excluded. Cases were categorized according to the first TD strategy selected for lesion crossing as the TD-primary IPT or TD-primary ADR group. The primary procedural outcome analysis was performed in the overall cohort defined by the initially selected TD strategy. The secondary procedural metrics were evaluated in cases that achieved both successful TD-based lesion crossing and final procedural success. The study was approved by the Central Medical Ethics Committee of Sakurabashi Watanabe Advanced Healthcare Hospital, Osaka, Japan (approval #26-07).

### Data collection

Baseline demographic and clinical data were obtained from the electronic medical records, including age, sex, cardiovascular risk factors (hypertension, diabetes mellitus, dyslipidemia, and smoking status), renal function and hemodialysis status, left ventricular ejection fraction, prior coronary artery bypass grafting, and relevant medications when available. Left ventricular ejection fraction was assessed using the 2-dimensional modified Simpson method. Lesion- and procedure-related variables were extracted from coronary angiography and IVUS images and were adjudicated by manual review by experienced operators.

### Study definitions

CTO was defined as a lesion with TIMI flow grade 0 and an estimated occlusion duration of ≥3 months. Lesion complexity was assessed using the J-CTO score (Multicenter CTO Registry in Japan)^10^ and the CASTLE (CABG, age, stump anatomy, tortuosity, length of occlusion, and extent of calcification) score (EuroCTO Registry).^11^ Angiographic classifications (eg, CTO entry type, calcification, bending, and lesion length) were applied as previously reported. Severe calcification was defined as calcification meeting either of the following criteria: 1) calcification arc ≥270°; or 2) calcified lesion length ≥5 mm. An ostial lesion was defined as a CTO with the proximal cap located at the ostium of the target vessel. Distal bifurcation was defined as a lesion with a side branch at the CTO exit site.

Procedural success was defined as successful crossing of the occlusion with both a guidewire and a balloon, adequate dilation of the occluded segment, and achievement of antegrade flow with <50% residual stenosis on the final angiogram. Total procedure time was defined as the time from the start of the procedure to the final angiogram. Lesion crossing time was defined as the time from when the guidewire approached the CTO entry on fluoroscopy to the time of true-lumen crossing. TD time was defined as the time from initiation of TD on angiography or IVUS to true-lumen crossing. Stent length was defined as the total stent length used to treat the CTO segment; cases completed without stent implantation, including those treated with balloon angioplasty alone or a drug-coated balloon, were assigned a stent length of 0 mm. Major adverse cardiac and cerebrovascular events were defined as a composite of cardiac death, noncardiac death, myocardial infarction, emergent target-vessel revascularization, and stroke. Only procedural outcomes and in-hospital events were evaluated. Postdischarge clinical follow-up was not analyzed in this study.

### Statistical analysis

Continuous variables are reported as median (IQR), and categorical variables as counts (percentages). Normality was assessed using Q-Q plots or the Shapiro-Wilk test. Univariate comparisons were performed between the TD-primary IPT and TD-primary ADR groups. Continuous variables were compared using the unpaired Student’s *t*-test for normally distributed data or the Mann-Whitney *U* test for non-normally distributed data. Categorical variables were compared using the chi-square test or Fisher exact test, as appropriate. Exact binomial 95% CIs were calculated for categorical procedural outcome rates. *P* values were not calculated for outcomes with zero events in both groups. Missing data were assessed for all variables included in the analyses. No missing data were present within each prespecified analysis population. Variables that were not clinically or procedurally defined in unsuccessful cases were not considered missing. Therefore, different denominators across analyses reflected prespecified analysis populations rather than missing data. No imputation was performed, and formal testing for missing completely at random was not applicable.

Because lesion crossing time and stent length differed significantly between groups in secondary analyses, exploratory multivariable linear regression analyses were performed for these 2 outcomes using forced-entry models with prespecified covariates. Covariates were selected a priori based on clinical relevance to TD-based wiring and CTO body complexity among components of the J-CTO score and included the initially selected TD strategy (TD-primary ADR vs TD-primary IPT), severe calcification, bending ≥45°, and occlusion length ≥20 mm. All prespecified covariates were entered as binary variables. Adjusted R^2^ values were reported to describe model fit. Assumptions for multivariable linear regression models were assessed using variance inflation factors to evaluate multicollinearity, residual-versus-fitted plots to assess homoscedasticity, and Q-Q plots and the Shapiro-Wilk test to assess normality of residuals. When residual normality was not satisfied, a sensitivity analysis using the log-transformed outcome was performed. A sensitivity analysis excluding cases with crossover from TD-IPT to TD-ADR was performed for secondary procedural metrics. A 2-sided *P* value <0.05 was considered statistically significant. Statistical analyses were performed using JMP, version 19.1.1 (SAS Institute Inc).

## Results

### Study cohort and baseline characteristics

The patient selection process is shown in Figure 1, and the TD strategy pathways and key findings are summarized in the Central Illustration. During the study period, CTO-PCI was performed in 346 cases (354 lesions). The TD strategy was attempted in 77 cases (80 lesions). Four cases involving treatment of multiple CTO lesions within a single session (7 lesions) were excluded from the per-case analyses; therefore, the main analytical cohort consisted of 73 cases (73 lesions). These 73 cases were categorized into the TD-primary IPT group or the TD-primary ADR group according to the initial TD strategy selected for lesion crossing.

**Figure 1:**
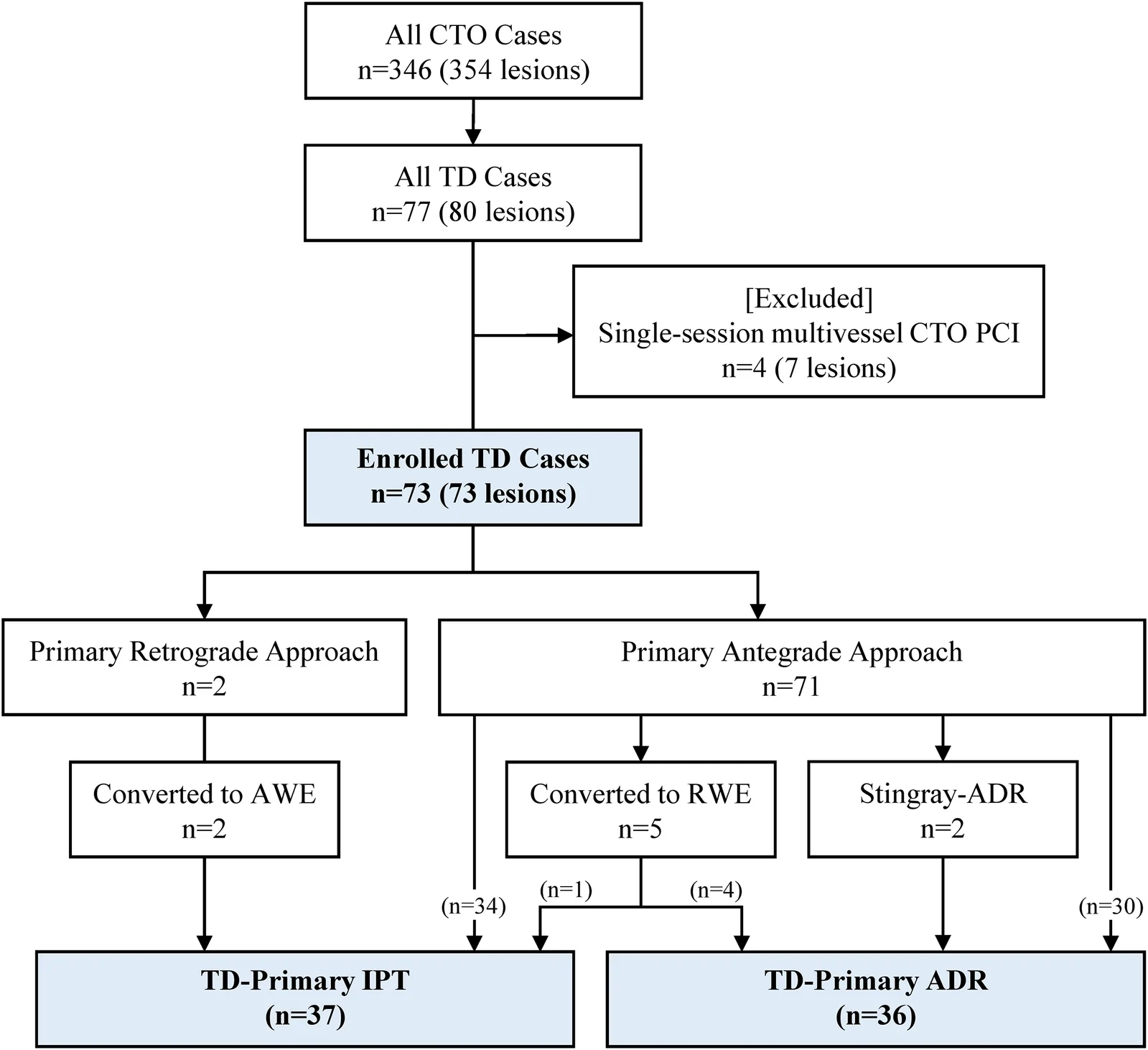
Study Population Flowchart Flowchart illustrating the selection of the study population. ADR = antegrade dissection and re-entry; AWE = antegrade wire escalation; CTO = chronic total occlusion; IPT = intraplaque tracking; PCI = percutaneous coronary intervention; RWE = retrograde wire escalation; TD = tip detection.

**Central Illustration:**
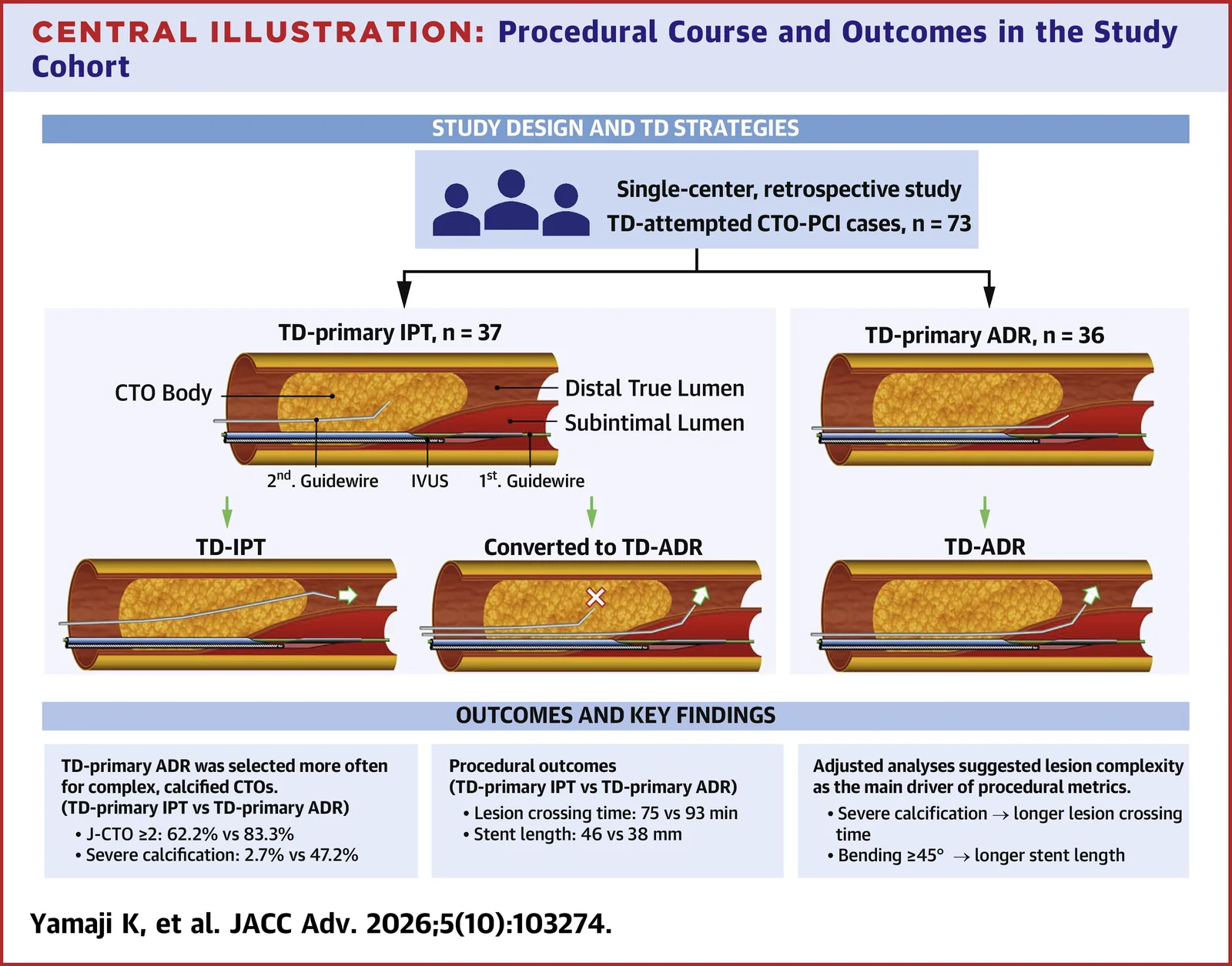
Procedural Course and Outcomes in the Study Cohort This figure summarizes the procedural course and outcomes in this study cohort. Patients were classified according to the initially selected TD strategy as TD-primary IPT or TD-primary ADR. In TD-IPT, when the first guidewire has entered the subintimal lumen, IVUS is advanced over that wire, and a second guidewire is directed by plaque tracking into the distal true lumen. In TD-ADR, IVUS-guided puncture is performed from the subintimal lumen toward the distal true lumen to achieve antegrade dissection and re-entry. Conversion from TD-IPT to TD-ADR was performed when distal true-lumen crossing was not achieved with TD-IPT. TD-primary ADR was selected more frequently in cases with complex, calcified CTOs in this cohort. In exploratory adjusted analyses, severe calcification was associated with longer lesion crossing time, and bending ≥45° was associated with longer stent length. IVUS = intravascular ultrasound; other abbreviations as in Figure 1.

Figure 2 shows the procedural course after this classification, including subsequent strategy changes and final procedural success. Of the 73 cases, 69 achieved both TD-based lesion crossing and final procedural success and were used for secondary analyses of procedural metrics.

**Figure 2:**
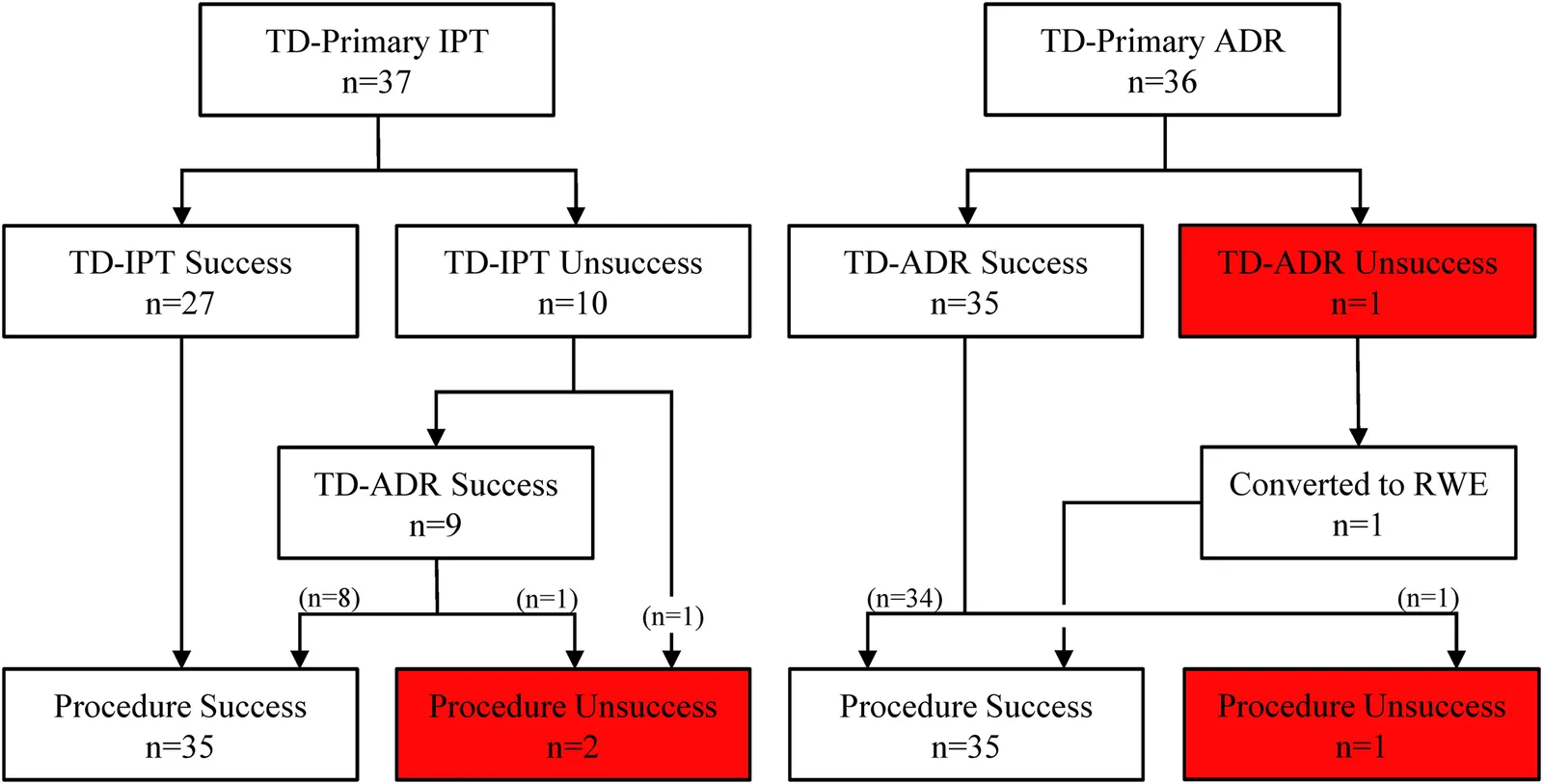
Procedural Flow of Tip Detection-Primary Intraplaque Tracking and Tip Detection-Primary Antegrade Dissection and Re-Entry Flowchart illustrating the procedural course after TD-primary IPT or TD-primary ADR. Cases with procedural failure or TD failure were excluded from secondary analyses of successful TD cases (red-shaded boxes; n = 4). Abbreviations as in Figure 1.

Figure 3 summarizes lesion-level outcomes across all 354 CTO lesions, including the distribution of final lesion-crossing strategies among successful cases. Overall procedural success for all CTO lesions was 97.5% (345/354; 95% CI: 95.2%-98.8%). Among the 9 unsuccessful cases, the final attempted strategy was TD in 3 cases, antegrade wire escalation in 5 cases, and retrograde wire escalation (RWE) in 1 case. In Figure 3, lesions crossed with TD (n = 76) comprise 69 lesions from the per-case analytical cohort with both successful TD-based lesion crossing and final procedural success, plus 7 lesions from the excluded multi-CTO-session cases.

**Figure 3:**
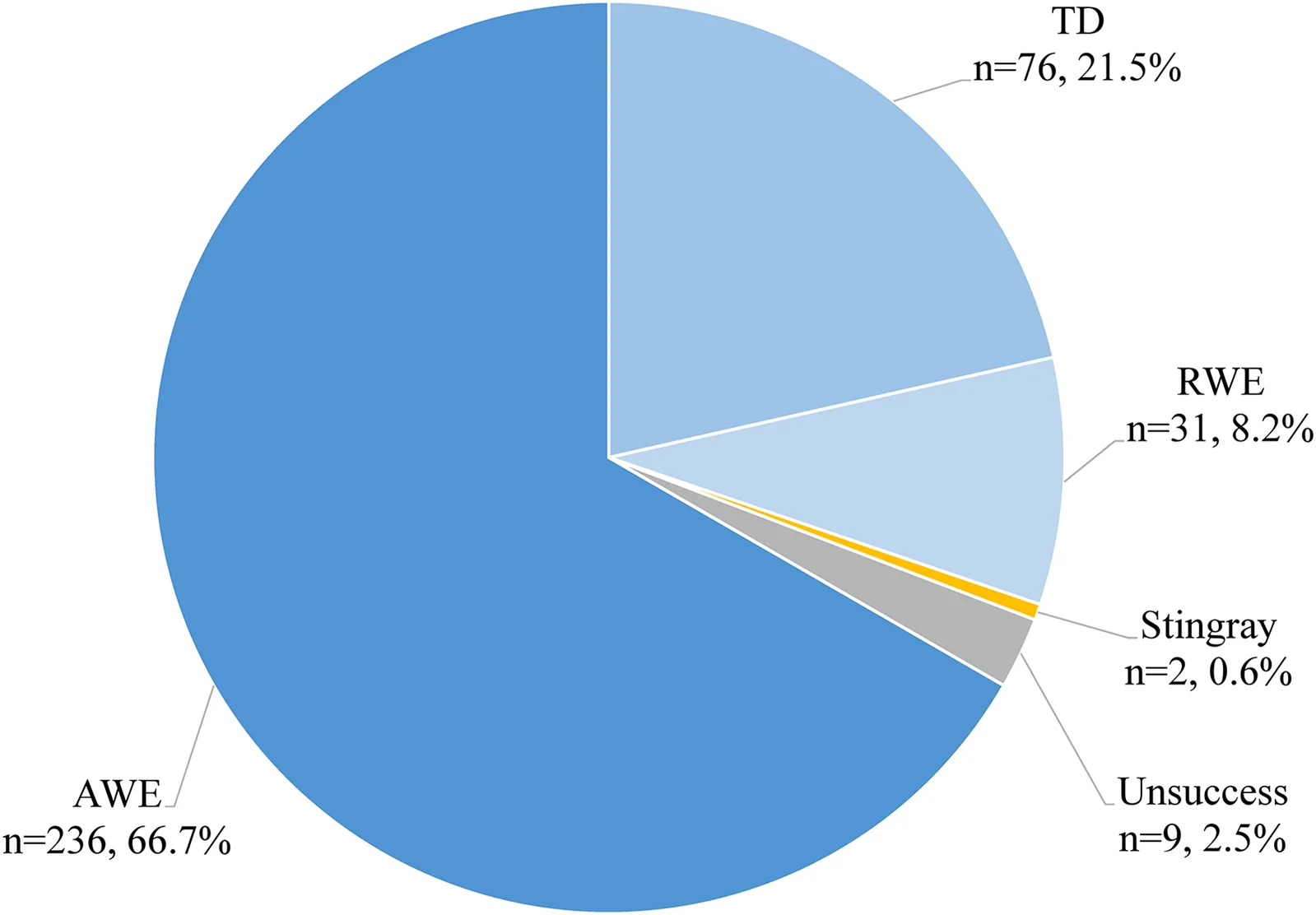
Distribution of Lesion-Crossing Strategies Final lesion-crossing strategies used in all 354 CTO lesions. Abbreviations as in Figure 1.

Patient and lesion characteristics are shown in Table 1. Baseline characteristics, including age, sex, and prevalence of coronary risk factors, did not differ significantly between groups, and target vessels were similarly distributed. Regarding lesion characteristics, the proportion of lesions with a J-CTO score ≥2 was significantly lower in the TD-primary IPT group than in the TD-primary ADR group (62.2% [23/37] vs 83.3% [30/36], *P* = 0.043). Among components of the J-CTO score, calcification was significantly more frequent in the TD-primary ADR group (27.0% [10/37] vs 63.9% [23/36], *P* = 0.002). In particular, severe calcification was rare in the TD-primary IPT group (2.7% [1/37] vs 47.2% [17/36], *P* < 0.001). Occlusion length did not differ significantly between groups (22 mm [15-35] vs 22 mm [15.8-25], *P* = 0.343). The proportion of lesions with a CASTLE score ≥4 did not differ significantly between groups (5.4% [2/37] vs 13.9% [5/36], *P* = 0.261).

**Table 1:** Demographic and Angiographic Characteristics

	TD-Primary IPT (n = 37)	TD-Primary ADR (n = 36)	Total (N = 73)	*P* Value
Age, y	61 (55.5-76)	64 (56-74)	62 (56-75.5)	0.960
Male	33 (89.2)	32 (88.9)	65 (89.0)	1.000
CABG history	1 (2.7)	1 (2.8)	2 (2.7)	1.000
Low LVEF (≤35%)	5 (13.5)	7 (19.4)	12 (16.4)	0.543
CKD (eGFR<35)	5 (13.5)	4 (11.1)	9 (12.3)	1.000
Dialysis	1 (2.7)	2 (5.6)	3 (4.1)	0.615
Dyslipidemia	23 (62.2)	30 (83.3)	53 (72.6)	0.065
Diabetes	16 (43.2)	18 (50.0)	34 (46.6)	0.642
Hypertension	28 (75.7)	30 (83.3)	58 (79.5)	0.564
Smoking	18 (48.7)	20 (55.6)	38 (52.1)	0.642
Target lesion				
RCA	15 (40.5)	12 (33.3)	27 (37.0)	0.630
LAD	11 (29.7)	7 (19.4)	18 (24.7)	0.417
LCx	9 (24.3)	14 (38.9)	23 (31.5)	0.214
Other	2 (5.4)	3 (8.3)	5 (6.9)	0.674
J-CTO score	2 (1-3)	2 (2-3)	2 (1-3)	0.138
0	2 (5.4)	1 (2.8)	3 (4.1)	1.000
1	12 (32.4)	4 (11.1)	16 (21.9)	0.046
2	11 (29.7)	19 (52.8)	30 (41.1)	0.045
3	10 (27.0)	7 (19.4)	17 (23.3)	0.444
4	2 (5.4)	4 (11.1)	6 (8.2)	0.430
5	0 (0)	1 (2.8)	1 (1.4)	0.493
≥2	23 (62.2)	30 (83.3)	53 (72.6)	0.043
CASTLE score	2 (1-3)	2 (2-3)	2 (2-3)	0.603
≥4	2 (5.4)	5 (13.9)	7 (9.6)	0.261
Blunt	23 (62.2)	24 (66.7)	47 (64.4)	0.808
Calcification	10 (27.0)	23 (63.9)	33 (45.2)	0.002
Severe calcification	1 (2.7)	17 (47.2)	18 (24.7)	<0.001
Bending ≥45°	11 (29.7)	8 (22.2)	19 (26.0)	0.595
Occlusion length	22 (15-35)	22 (15.8-25)	22 (15-30)	0.343
Occlusion length ≥20 mm	23 (62.2)	21 (58.3)	44 (60.3)	0.813
Re-try lesion	5 (13.5)	9 (25.0)	14 (19.2)	0.247
In-stent lesion	2 (5.4)	2 (5.6)	4 (5.5)	1.000
Ostium lesion	9 (24.3)	10 (27.8)	19 (26.0)	0.794
Distal bifurcation	16 (43.2)	14 (38.9)	30 (41.1)	0.813

Values are median (IQR) or n (%). Baseline characteristics are shown according to the initially selected TD strategy.

ADR = antegrade dissection and re-entry; CABG = coronary artery bypass grafting; CKD = chronic kidney disease; CTO = chronic total occlusion; eGFR = estimated glomerular filtration rate; IPT = intraplaque tracking; LAD = left anterior descending artery; LCx = left circumflex artery; LVEF = left ventricular ejection fraction; RCA = right coronary artery; TD = tip detection.

### Procedural course and outcomes

Among the 73 cases, the rates of TD-based lesion crossing and procedural success were 97.3% (36/37; 95% CI: 85.8%-99.9%) and 94.6% (35/37; 95% CI: 81.8%-99.3%) in the TD-primary IPT group, and 97.2% (35/36; 95% CI: 85.5%-99.9%) and 97.2% (35/36; 95% CI: 85.5%-99.9%) in the TD-primary ADR group, respectively. As shown in Figure 2, among the 70 cases with final procedural success, 1 case required conversion to RWE after unsuccessful TD. Lesion crossing was achieved with TD-IPT in 27 cases in the TD-primary IPT group. Ten cases could not be crossed with TD-IPT; 9 were converted to TD-ADR and achieved lesion crossing, whereas the remaining 1 case was ultimately classified as a procedural failure without crossover. Table 2 summarizes procedural details of these 9 cases. In 1 converted case, the guidewire could not be advanced into the major side branch, and the procedure was classified as unsuccessful.

**Table 2:** Lesion Characteristics and Procedural Outcomes of Cases Converted From TD-Primary IPT to TD-ADR

Patient #	Target Lesion	J-CTO Score	Procedure Success	TD-ADR Success	Total Procedure Time, min	Lesion Crossing Time, min	TD Time, min	
							TD-IPT	TD-ADR
1	RCA	3	Success	Success	100	52	27	3
2	RCA	2	Success	Success	157	121	67	18
3	LCx	1	Success	Success	326	183	57	10
4	RCA	3	Success	Success	189	126	12	68
5	LAD	1	Success	Success	130	76	52	3
6	RCA	3	Success	Success	228	183	79	55
7	LCx	2	Success	Success	219	149	40	69
8	LAD	1	Success	Success	97	50	22	13
9	LCx	1	Failure	Success	227	151	24	84

This table summarizes procedural components in 9 cases in which the approach was initiated with TD-IPT and subsequently converted to TD-ADR. TD time is presented separately as TD-IPT time and TD-ADR time.

Abbreviations as in Table 1.

In the TD-primary ADR group, TD failed in 1 case, and the strategy was converted to RWE, resulting in procedural success. In another case, TD achieved lesion crossing, but the guidewire subsequently slipped out; the operator treated another stenotic lesion and terminated the session, and CTO-PCI was classified as unsuccessful. No cases in the TD-primary ADR group used TD-IPT in combination.

Table 3 shows procedural outcomes in the overall cohort according to the initially selected TD strategy. Procedural success and TD success were similarly high in both groups. Crossover from TD to another TD strategy occurred in 9 cases in the TD-primary IPT group and in no cases in the TD-primary ADR group; all such crossovers represented conversion from TD-IPT to TD-ADR. Crossover from TD to RWE occurred in 1 case in the TD-primary ADR group. Total procedure time and fluoroscopy time tended to be longer in the TD-primary ADR group, although the differences were not statistically significant. Radiation dose was lower in the TD-primary IPT group (2,139 mGy [1,573.5-4,091] vs 3,235.5 mGy [2,270.8-5,185.8], *P* = 0.034), whereas contrast volume did not differ between groups. No in-hospital major adverse cardiac and cerebrovascular events occurred. In each group, 2 cases of guidewire-related coronary perforation were observed (5.4% [2/37; 95% CI: 0.7%-18.2%] vs 5.6% [2/36; 95% CI: 0.7%-18.7%]); however, all events were minor and were successfully managed with balloon tamponade or thromboembolization.

**Table 3:** Procedural Outcomes, MACCE, and Complications

	TD-Primary IPT (n = 37)		TD-Primary ADR (n = 36)		Total (N = 73)		*P* Value
	Value	95% CI	Value	95% CI	Value	95% CI	
Procedural success	35 (94.6)	81.8-99.3	35 (97.2)	85.5-99.9	70 (95.9)	88.5-99.1	1.000
TD success	36 (97.3)	85.8-99.9	35 (97.2)	85.5-99.9	71 (97.3)	90.5-99.7	1.000
Transfemoral approach	34 (91.9)	78.1-98.3	36 (100)	90.3-100	70 (95.9)	88.5-99.1	0.240
RWE before TD	3 (8.1)	1.7-21.0	3 (8.3)	1.8-22.5	6 (8.2)	3.1-17.0	1.000
Stingray-ADR before TD	0 (0)	0.0-9.5	2 (5.6)	0.7-18.7	2 (2.7)	0.3-9.5	0.240
Crossover from TD to RWE	0 (0)	0.0-9.5	1 (2.8)	0.1-14.5	1 (1.4)	0.0-7.4	0.493
Crossover from TD to another TD strategy	9 (24.3)	11.8-41.2	0 (0)	0.0-9.7	9 (12.3)	5.8-22.1	0.002
Total procedure time, min	138 (114-204)	—	165 (133-217)	—	157 (123.5-215)	—	0.121
Fluoroscopy time, min	72 (58.7-104.5)	—	96.3 (66.2-116.2)	—	86 (61.5-111.7)	—	0.062
Radiation dose, mGy	2,139 (1,573.5-4,091)	—	3,235.5 (2,270.8-5,185.8)	—	2,892 (2061-4,344.5)	—	0.034
Contrast volume, ml	120 (85-155)	—	120 (90-157.5)	—	120 (90-155)	—	0.921
MACCE							
Cardiac death	0 (0)	0.0-9.5	0 (0)	0.0-9.7	0 (0)	0.0-4.9	—
Noncardiac death	0 (0)	0.0-9.5	0 (0)	0.0-9.7	0 (0)	0.0-4.9	—
Myocardial infarction	0 (0)	0.0-9.5	0 (0)	0.0-9.7	0 (0)	0.0-4.9	—
Emergent target vessel revascularization	0 (0)	0.0-9.5	0 (0)	0.0-9.7	0 (0)	0.0-4.9	—
Stroke	0 (0)	0.0-9.5	0 (0)	0.0-9.7	0 (0)	0.0-4.9	—
Procedural complications							
Guidewire-related vessel perforation	2 (5.4)	0.7-18.2	2 (5.6)	0.7-18.7	4 (5.5)	1.5-13.4	1.000
Cardiac tamponade	0 (0)	0.0-9.5	0 (0)	0.0-9.7	0 (0)	0.0-4.9	—

Values are median (IQR) or n (%). Exact binomial 95% CIs were calculated for categorical procedural outcome rates only. *P* values were not calculated for outcomes with zero events in both groups. Procedural outcomes are reported in the overall cohort according to the initially selected TD strategy. All crossovers from TD to another TD strategy occurred from TD-IPT to TD-ADR.

MACCE = major adverse cardiac and cerebrovascular events; RWE = retrograde wire escalation; other abbreviations as in Table 1.

Table 4 shows secondary procedural metrics among 69 cases with successful TD-based lesion crossing and final procedural success. Lesion crossing time was significantly shorter in the TD-primary IPT group (75 minutes [51-108] vs 93 minutes [63-137.3], *P* = 0.046), whereas total procedure time and TD time did not differ significantly between groups. Stent length was shorter in the TD-primary ADR group in univariable analysis (38 mm [28-54] vs 46 mm [38-64], *P* = 0.041). In the sensitivity analysis excluding TD-IPT to TD-ADR crossover, the direction of these findings was generally similar.

**Table 4:** Secondary Procedural Metrics in Successful TD Cases

Successful TD Cases	TD-Primary IPT (n = 35)	TD-Primary ADR (n = 34)	Total (N = 69)	*P* Value
Total procedure time, min	135 (108-189)	167.5 (135.3-221.0)	150 (122.5-209.0)	0.055
Total lesion crossing time, min	75 (51-108)	93 (63-137.3)	82 (56-125)	0.046
TD time, min	17 (5-50)	16.5 (7-44)	17 (6-46)	0.782
Stent length, mm	46 (38-64)	38 (28-54)	44 (30-57)	0.041

**Excluding TD-IPT to TD-ADRCrossoverCases**	**(n = 27)**	**(n = 34)**	**(N= 61)**	

Total procedure time, min	132 (108-172)	167.5 (135.3-221.0)	150 (122.5-200)	0.026
Total lesion crossing time, min	68 (51-87)	93 (63.0-137.3)	81 (56-116)	0.009
TD time, min	8 (5-33)	16.5 (7-44)	14 (5.5-38.5)	0.097
Stent length, mm	46 (38-64)	38 (28-54)	44 (31.5-57)	0.041

Values are median (IQR). Secondary procedural metrics were assessed in cases with successful TD-based lesion crossing and final procedural success. The sensitivity analysis excluded cases with crossover from TD-IPT to TD-ADR. These analyses should be interpreted as exploratory.

Abbreviations as in Table 1.

In an exploratory analysis within the TD-primary IPT group, successful initial TD-IPT cases (n = 27) were compared with cases requiring crossover to TD-ADR or resulting in TD/procedural failure (n = 10). Target vessel distribution and individual J-CTO score components did not differ significantly between groups. Among clinical variables, diabetes and dyslipidemia were less frequent in successful initial TD-IPT cases, whereas severe calcification was observed in only 1 TD-primary IPT case. These findings should be interpreted cautiously because of the small sample size and multiple exploratory comparisons. Detailed results are shown in Supplemental Table 1.

Table 5 summarizes device use in cases with successful TD-based lesion crossing and final procedural success. Microcatheter utilization was similar between the TD-primary IPT and TD-primary ADR groups, and the Corsair Pro microcatheter (Asahi Intecc) was the most frequently used (46/69 [66.7%]). In recent practice, Caravel MC (Asahi Intecc) is increasingly used when the false-lumen space is narrow; however, its use during the study period remained limited (3/69 [4.4%]). Zizai (Terumo) and Corsair Pro XS (Asahi Intecc) were each used in one non-calcified CTO case in the TD-primary IPT group, but because of insufficient backup support, these devices are currently less commonly used for TD. Regarding guidewires, several Conquest-series guidewires were used in successful TD-IPT cases, whereas Conquest Pro 12ST (Asahi Intecc) was predominantly used for TD-based lesion crossing in cases converted to TD-ADR and in successful TD-primary ADR cases. Because ADR requires high penetration performance with pinpoint accuracy at the target point, this guidewire may have been selected more frequently. IVUS was performed using AnteOwl WR (Terumo) in >90% of cases; the remaining cases used AltaView (Terumo).

**Table 5:** Devices Used in Successful Procedures

	TD-Primary IPT (n = 35)		Successful TD-Primary ADR (n = 34)	Total (N = 69)
	Successful TD-IPT (n = 27)	Converted to TD-ADR (n = 8)		
8-F guiding catheter	24 (88.9)	5 (62.5)	31 (91.2)	60 (87.0)
Microcatheter used in TD	-	-	-	-
Corsair Pro	21 (77.8)	3 (37.5)	22 (64.7)	46 (66.7)
Finecross	4 (14.8)	3 (37.5)	11 (32.4)	18 (26.1)
Caravel MC	0 (0.0)	2 (25.0)	1 (2.9)	3 (4.4)
Others	2 (7.4)	0 (0.0)	0 (0)	2 (2.9)
Guidewire used in TD	-	-	-	-
Conquest Pro	8 (29.6)	0 (0.0)	0 (0)	8 (11.6)
Conquest Pro 12g	4 (14.8)	1 (12.5)	1 (2.9)	6 (8.7)
Conquest Pro 12ST	5 (18.5)	6 (75.0)	31 (91.2)	42 (60.9)
Conquest Pro 8-20	6 (22.2)	1 (12.5)	2 (5.9)	9 (13.0)
Others	4 (14.8)	0 (0.0)	0 (0)	4 (5.8)
AnteOwl WR IVUS	27 (100.0)	7 (87.5)	31 (91.2)	65 (94.2)

Values are n (%). Device use was evaluated in cases with successful TD-based lesion crossing and final procedural success. Converted to TD-ADR indicates crossover from initial TD-IPT to TD-ADR. Guidewires indicate those used for TD-based lesion crossing.

IVUS = intravascular ultrasound; other abbreviations as in Table 1.

In all cases, the guidewire had entered the subintimal space during antegrade wire escalation or angiography-guided 3-dimensional wiring. Dedicated intentional subintimal entry techniques, such as BASE or scratch-and-go, were not used in this cohort.

### Multivariable analyses

Exploratory multivariable linear regression analyses using forced-entry models with prespecified covariates were performed for lesion crossing time and stent length (Table 6). The initially selected TD strategy was not significantly associated with lesion crossing time (β = 4.0; SE = 7.3; *P* = 0.585; 95% CI: −10.6 to 18.7), whereas severe calcification was associated with longer lesion crossing time (β = 23.6; SE = 8.5; *P* = 0.007; 95% CI: 6.6-40.5). For stent length, bending ≥45° was significantly associated with longer stent length (β = 8.9; SE = 2.6; *P* = 0.001; 95% CI: 3.6-14.2). TD-primary ADR showed a nonsignificant trend toward shorter stent length after adjustment (β = −5.2; SE = 2.8; *P* = 0.063; 95% CI: −10.7 to 0.3). Because of the limited sample size and imbalance in lesion complexity between groups, multivariable models were considered exploratory. The adjusted R^2^ values were 0.130 for the lesion crossing time model and 0.207 for the stent length model, indicating that the prespecified covariates explained only a modest proportion of variability in these procedural metrics. No substantial multicollinearity was observed, with all variance inflation factors <1.4. Residual diagnostics for the stent length model did not show major violations of model assumptions. Because residual normality was not satisfied in the lesion crossing time model, a sensitivity analysis using log-transformed lesion crossing time was performed and yielded findings consistent with the primary model.

**Table 6:** Exploratory Multivariable Linear Regression Analysis for Lesion Crossing Time and Stent Length

	β	SE	*P* Value	95% CI
Lesion crossing time				
TD-primary ADR	4.0	7.3	0.585	−10.6 to 18.7
Severe calcification	23.6	8.5	0.007	6.6-40.5
Bending ≥45°	−4.1	7.0	0.558	−18.2 to 9.9
Occlusion length ≥20 mm	3.9	6.4	0.540	−8.8 to 16.6
Stent length				
TD-primary ADR	−5.2	2.8	0.063	−10.7 to 0.3
Severe calcification	1.8	3.2	0.581	−4.6 to 8.2
Bending ≥45°	8.9	2.6	0.001	3.6-14.2
Occlusion length ≥20 mm	4.5	2.4	0.067	−0.3 to 9.2

Exploratory multivariable linear regression analyses were performed using forced-entry models with prespecified covariates. Covariates included the initially selected TD strategy, severe calcification, bending ≥45°, and occlusion length ≥20 mm. For the initially selected TD strategy, TD-primary IPT was used as the reference category. Values are expressed as regression coefficients (β), standard errors (SE), *P* values, and 95% CIs. Variables with *P* < 0.05 were considered statistically significant. Adjusted R^2^ values were 0.130 and 0.207 for the lesion crossing time and stent length models, respectively.

Abbreviations as in Table 1.

## Discussion

### Principal findings

TD-based lesion crossing was highly successful in both groups, with TD success rates of 97.3% in the TD-primary IPT group and 97.2% in the TD-primary ADR group. Final procedural success was also high (94.6% and 97.2%, respectively), suggesting that both TD strategies can be performed with a high likelihood of technical success in appropriately selected cases. Notably, in the TD-primary IPT group, 9 cases required conversion to TD-ADR, requiring additional procedural time. TD-based lesion crossing was ultimately achieved in all 9 converted cases; however, 1 case did not result in final procedural success. These findings indicate that TD-ADR may serve not only as an initially selected strategy but also as a bailout option after unsuccessful TD-IPT.

The study population included 2 cases in which Stingray-ADR failed but lesion crossing was achieved using TD-ADR. Additionally, 5 cases involved failed retrograde attempts; among these, lesion crossing was achieved with TD-IPT in 3 cases and with TD-ADR in 2 cases. These findings support prior reports suggesting that the TD method has a higher success rate than the Stingray-ADR and may be effective even in cases where a retrograde approach is difficult.^7^^,^^12^ Conversely, there was 1 case in which lesion crossing was achieved only after conversion to a retrograde strategy following failure of TD-ADR, underscoring the importance of selecting an appropriate approach tailored to each case. Because the initial TD strategy was selected at operator discretion, this study should not be interpreted as a direct comparison of the intrinsic efficacy of TD-IPT vs TD-ADR. Rather, the findings should be viewed as an exploratory description of lesion-based strategy selection and procedural outcomes in real-world TD-guided CTO-PCI.

### Selection of TD-IPT vs TD-ADR based on patient characteristics and lesion morphology

Univariate analyses suggested that TD-ADR was more frequently selected for complex lesions such as J-CTO score ≥2, and the major driver of higher J-CTO scores in this context was the presence of calcification. This may reflect the fact that calcification substantially limits IVUS visualization, making identification of the false-lumen entry point and subsequent plaque tracking during TD-IPT more difficult, thereby prompting operators to select TD-ADR. When calcium exists between the true lumen and the false lumen, the guidewire can become difficult to visualize once it passes behind the calcified segment, even if entry into the lesion is initially observed by IVUS. In such cases, operators may need to wait for the guidewire to reappear at a more distal point where visualization becomes possible. During this interval, the guidewire must be advanced under fluoroscopy, and an important technical point is to “fuse” angiographic and IVUS images—that is, to rotate the IVUS image so that the guidewire tip direction is aligned between imaging modalities while referencing angiography during advancement behind the calcified segment. In this situation, the second wire should be advanced with its tip directed toward the IVUS catheter under fluoroscopic guidance. This maneuver allows the second wire to track along the vessel wall, shielded by the calcification, and navigate into the true lumen. However, during guidewire passage through the calcified segment, there is a risk of guidewire exit outside the vessel or re-entry into another subintimal space. Therefore, crossing the calcified segment within the subintimal space and returning to the true lumen using TD-ADR may be more reliable, which may explain why TD-ADR was more frequently selected for calcified CTOs. Severe calcification is reported to be a major cause of CTO-PCI failure.^13^^,^^14^ In our cohort, the overall procedural success rate was high (95.9% [70/73]), and lesion-crossing success with TD-ADR in calcified lesions was 100% (23/23). These findings suggest that TD-ADR may represent an effective solution for CTO lesions with severe calcification. From a broader complexity perspective, the distribution of CASTLE scores did not differ significantly between groups. Notably, however, all TD-primary IPT cases with a CASTLE score ≥4 ultimately required crossover to TD-ADR after unsuccessful TD-IPT and subsequently achieved TD-based lesion crossing, suggesting that TD-ADR may serve as a valuable contingency strategy in selected highly complex lesions. As shown in Supplemental Table 1, in the exploratory analysis within the TD-primary IPT group, target vessel distribution and individual J-CTO score components did not differ significantly between cases with successful initial TD-IPT and those requiring crossover to TD-ADR. Because overall TD failure was rare, no definitive vessel-specific conclusion regarding TD success could be drawn. These findings suggest that conventional angiographic CTO variables alone may not fully explain TD-IPT feasibility. Procedural-anatomic factors, including IVUS visualization, the spatial relationship between the subintimal lumen and distal true lumen, subintimal space enlargement, and the ability to direct the second guidewire into the plaque toward the distal true lumen, may also influence TD-IPT success.

### Selection of TD-IPT vs TD-ADR based on procedural outcomes

Regarding procedural outcomes, prespecified multivariable analyses did not identify the initially selected TD strategy as a significant determinant of lesion crossing time. Instead, severe calcification was associated with longer lesion crossing time. These findings suggest that procedural efficiency may be driven primarily by lesion complexity rather than by the specific TD strategy initially selected. Because lesion crossing time reflects the entire procedural sequence from guidewire approach to true-lumen crossing, it should not be interpreted as an isolated measure of TD maneuver performance. TD time, a more TD-specific procedural metric, did not differ significantly between groups; however, after excluding TD-IPT to TD-ADR crossover cases, TD time tended to be shorter in the TD-primary IPT group. This observation may partly reflect the preferential selection of TD-primary ADR for lesions in which TD-IPT was considered less suitable, such as severely calcified lesions.

In successful TD cases, stent length was shorter in the TD-primary ADR group in univariable analysis. However, after adjustment using prespecified covariates, TD-primary ADR showed only a nonsignificant trend toward shorter stent length, whereas bending ≥45° was significantly associated with longer stent length. Therefore, the observed difference in stent length should be interpreted as exploratory. When interpreting these secondary procedural metrics, it should be noted that lesion crossing time, TD time, and stent length were evaluated only in successful TD cases because these metrics are inherently undefined in cases without successful TD-based lesion crossing and final procedural success. Accordingly, the different denominators across analyses reflected prespecified analysis populations rather than missing observations. Unlike conventional Stingray-ADR, TD-ADR may enable more accurate re-entry into the true lumen at the intended target point and may reduce unnecessary subintimal extension in selected cases. Conversely, TD-IPT may allow lesion crossing while maintaining a more intraplaque course through the CTO segment and may therefore be advantageous when plaque tracking is technically feasible. These findings suggest that TD-IPT can achieve comparable TD-related procedural performance in selected lesions. Therefore, rather than favoring TD-ADR uniformly, it may be reasonable to first assess the feasibility of TD-IPT based on calcification severity, IVUS visualization, and the anticipated plaque-tracking route. TD-ADR may be selected when TD-IPT is considered technically challenging, such as in severely calcified lesions with limited effective IVUS guidance, or may serve as a bailout strategy after unsuccessful TD-IPT. Accordingly, careful lesion assessment and individualized selection of TD-IPT vs TD-ADR may be essential.

### Study limitations

This study has several limitations. First, this was a single-center retrospective study with a limited sample size. Second, selection of TD-IPT vs TD-ADR was left to operator discretion so that the present findings should not be interpreted as demonstrating the superiority of either strategy. Third, operators were not standardized across cases. Although physicians experienced in TD techniques were involved, interoperator differences in skill may have influenced TD time, lesion crossing time, and total procedure time. Multicenter prospective studies with standardized operator criteria are warranted for more accurate evaluation. Fourth, secondary procedural metrics, including lesion crossing time, TD time, and stent length, were evaluated only in successful TD cases and should therefore be interpreted as exploratory. Fifth, crossover from TD-IPT to TD-ADR may have influenced procedural metrics in the TD-primary IPT group, although a sensitivity analysis excluding TD-IPT to TD-ADR crossover was performed. Sixth, the multivariable models were limited by the small sample size despite restricting the number of prespecified covariates. Lesion crossing time and stent length may be influenced by unmeasured procedural and anatomical factors, including operator technique, IVUS visibility, guidewire controllability, and device support. Finally, postdischarge clinical follow-up was not analyzed; therefore, long-term clinical outcomes, including restenosis, repeat revascularization, myocardial infarction, and survival, were not assessed.

## Conclusions

This single-center retrospective study compared factors influencing the choice between TD-primary IPT and TD-primary ADR and evaluated their procedural outcomes. TD-primary ADR was preferentially selected for complex lesions, particularly those with severe calcification. In exploratory multivariable analyses, severe calcification was associated with longer lesion crossing time, whereas the initially selected TD strategy was not an independent determinant of lesion crossing time. These findings suggest that TD-IPT should be considered when technically feasible based on lesion morphology, IVUS visualization, and the anticipated plaque-tracking route, whereas TD-ADR may be appropriate when TD-IPT appears challenging or as a bailout strategy after unsuccessful TD-IPT. Individualized TD strategy selection may help optimize CTO-PCI.

Perspectives **COMPETENCY IN PATIENT CARE AND PROCEDURAL SKILLS:** This study may help operators select between TD-IPT and TD-ADR during CTO-PCI by focusing on lesion morphology, calcification severity, IVUS visualization, and the anticipated plaque-tracking route. A lesion-based approach may support procedural planning and promote more consistent use of IVUS-guided antegrade approaches beyond specialized CTO centers.

**TRANSLATIONAL OUTLOOK:** To standardize TD strategy selection, future multicenter prospective studies are needed to validate this lesion-based approach across operators and centers and to determine its impact on procedural efficiency, safety, and long-term outcomes.

## Funding support and author disclosures

The authors have reported that they have no relationships relevant to the contents of this paper to disclose.
