# Supplementary material for: Strategy Selection and Outcomes of Tip-Detection in CTO-PCI: Intraplaque Tracking vs Antegrade Dissection and Re-Entry
Source: JACC Adv. 2026 Sep 22;5(10):103274. doi: 10.1016/j.jacadv.2026.103274 (PMC13634284; doi:10.1016/j.jacadv.2026.103274)
Supplement: Supplementary file 1 [file mmc1.docx]

**SUPPLEMENTAL Material**

**Supplemental Table 1. Demographic and Angiographic Characteristics According to Initial TD-IPT Success**

|  | **TD-primary IPT success (n=27)** | | **TD-primary IPT unsuccess (n=10)** | | **p-value** | |  |
| --- | --- | --- | --- | --- | --- | --- | --- |
| Age | 62 (56 - 76) | | 60 (54.4 - 76.5) | | 0.810 | |  |
| Male | 24 (88.9) | | 9 (90.0) | | 1.000 | |  |
| CABG history | 1 (3.7) | | 0 (0.0) | | 1.000 | |  |
| Low LVEF (≦35%) | 4 (14.8) | | 1 (10.0) | | 1.000 | |  |
| CKD (eGFR<35) | 5 (18.5) | | 0 (0.0) | | 0.295 | |  |
| Dialysis | 1 (2.7) | | 0 (0.0) | | 1.000 | |  |
| Dyslipidemia | 15 (55.6) | | 8 (80.0) | | 0.260 | |  |
| Diabetes | 8 (29.6) | | 8 (80.0) | | 0.010 | |  |
| Hypertension | 21 (78.8) | | 7 (70.0) | | 0.679 | |  |
| Smoking | 14 (51.9) | | 4 (40.0) | | 0.714 | |  |
| Target lesion |  | |  | |  | |  |
| RCA | 11 (40.7) | | 4 (40.0) | | 1.000 | |  |
| LAD | 8 (29.6) | | 3 (30.0) | | 1.000 | |  |
| LCx | 6 (22.2) | | 3 (30.0) | | 0.679 | |  |
| Other | 2 (7.4) | | 0 (0.0) | | 1.000 | |  |
| J-CTO Score | 2 (1 - 3) | | 2 (1 - 3) | | 0.817 | |  |
| 0 | 2 (7.4) | | 0 (0.0) | | 1.000 | |  |
| 1 | 9 (33.3) | | 3 (30.0) | | 1.000 | |  |
| 2 | 7 (25.9) | | 4 (40.0) | | 0.442 | |  |
| 3 | 7 (25.9) | | 3 (30.0) | | 1.000 | |  |
| 4 | 2 (7.4) | | 0 (0.0) | | 1.000 | |  |
| 5 | 0 (0) | | 0 (0) | | 1.000 | |  |
| ≥2 | 16 (59.3) | | 7 (70.0) | | 0.710 | |  |
| CASTLE score | 2 (1 - 3) | | 3 (2 - 3.3) | | 0.050 | |  |
| ≥4 | 0 (0.0) | | 2 (20.0) | | 0.068 | |  |
| Blunt | 17 (63.0) | | 6 (60.0) | | 1.000 | |  |
| Calcification | 8 (29.6) | | 2 (20.0) | | 0.694 | |  |
| Severe calcification | 0 (0.0) | | 1 (10.0) | | 0.270 | |  |
| Bending ≥45° | 7 (25.9) | | 4 (40.0) | | 0.442 | |  |
| Occlusion length | | 25 (15 - 35) | | 21.5 (12.3 - 38.3) | | 0.705 | |
| Occlusion length ≥20 mm | | 17 (63.0) | | 6 (60.0) | | 1.000 | |
| Re-try lesion | | 4 (14.8) | | 1 (10.0) | | 1.000 | |
| In-stent lesion | | 1 (3.7) | | 1 (10.0) | | 0.473 | |
| Ostium lesion | | 6 (22.2) | | 3 (30.0) | | 0.679 | |
| Distal bifurcation | | 12 (44.4) | | 2 (20.0) | | 0.260 | |

Values are presented as median (interquartile range) or numbers (percentages), as appropriate. Successful initial TD-IPT cases were compared with cases requiring crossover to TD-ADR or resulting in TD/procedural failure. ADR=antegrade dissection and re-entry; CABG=coronary artery bypass grafting; CKD=chronic kidney disease; CTO=chronic total occlusion; IPT=intra-plaque tracking; LAD=left anterior descending artery; LCx=left circumflex artery; LVEF=left ventricular ejection fraction; RCA=right coronary artery; TD=tip detection.
